# Integrating morphophysiological traits with salt-responsive gene expression uncovers cultivar-specific tolerance mechanisms in faba beans facing NaCl stress

**DOI:** 10.1038/s41598-026-51413-1

**Published:** 2026-05-10

**Authors:** Sobhi F. Lamlom, Asmaa S. A. Khalifa, Mohamed Abdelhamid, Amera F. M. Zaitoun, Nader R. Abdelsalam

**Affiliations:** 1 Department of Plant Production, Faculty of Agriculture Saba Basha, Alexandria University, Alexandria, Egypt; 2 Agricultural Botany Department, Faculty of Agriculture, Alexandria University, Saba Basha, 21531 Egypt

**Keywords:** *Vicia faba*, Salinity tolerance, Root architecture, *DREB2*, Epicuticular wax, Principal component analysis, Gene expression, Osmotic adjustment, Biotechnology, Plant sciences

## Abstract

Soil salinization constrains faba bean (*Vicia faba* L.) production across North Africa and the Near East. Three Egyptian cultivars, Nubaria 1, Giza 716, and Sakha 5, were subjected to 0, 150, and 200 mM NaCl under controlled greenhouse conditions. Twenty morphological, physiological, and biochemical traits were quantified, along with quantitative RT-PCR profiling of 11 salt-responsive genes. Two-way ANOVA detected significant effects of cultivar, NaCl treatment, and their interaction for a subset of traits, including root length, leaf K⁺ concentration, net photosynthesis, stomatal conductance, intercellular CO₂, and three epicuticular wax fractions, indicating that these traits responded differently to salinity across genotypes. For a separate subset branch number, root dry weight, root-to-shoot ratio, and leaf nitrogen, only the cultivar term was significant, with no detectable effect of treatment or interaction. Nubaria 1 showed the smallest reductions in shoot dry weight (14% at 200 mM NaCl), maintained leaf K⁺ concentration across all salinity levels (13.3–13.6 g kg⁻¹), and retained the highest pod number at 200 mM NaCl (1.78 pods per plant). It also showed the lowest fold induction across all 11 genes at both stress levels. Sakha 5 showed the largest increase in root length under stress, from 3,577 cm in the control to 8,664 cm at 200 mM NaCl (+ 142%), the highest fold-induction across all gene categories, and produced zero pods at 200 mM NaCl in all replicates. Giza 716 had the highest net photosynthetic rate under control conditions (10.33 µmol CO₂ m⁻² s⁻¹) and was intermediate for most traits across treatment levels. In all three cultivars, net photosynthesis declined, and intercellular CO₂ rose simultaneously with increasing NaCl, from 327 to 364 µmol mol⁻¹ under control to 378–413 µmol mol⁻¹ at 200 mM NaCl. Total epicuticular wax reached its maximum at 150 mM NaCl in all cultivars and declined at 200 mM NaCl in Sakha 5 and Giza 716, despite continued CER1 induction in Sakha 5. Principal component analysis explained 70.7% of total variance across two components (PC1: 39.5%; PC2: 31.2%), with cultivar samples remaining separated along PC1 across all treatment levels. Leaf K⁺ concentration and net photosynthesis under stress showed the most consistent differences among cultivars and are identified as practical traits for early-stage phenotyping in salt-tolerance screening programs.

**Supplementary Information:**

The online version contains supplementary material available at 10.1038/s41598-026-51413-1.

## Introduction

Secondary salinization is accelerating across the world’s irrigated landscapes, with current estimates placing approximately 20% of irrigated and 33% of total arable land under some degree of salt-induced degradation^1,2^. An estimated ten million hectares of productive agricultural land are lost annually due to poorly managed irrigation, rising water tables, and climate aridification^3^. For most glycophytic crops, this leads to high soil NaCl levels, reducing water availability and exposing plants to toxic ions, which disrupts cellular functions and consistently lowers yields^4^. As demand for protein-rich foods grows and saline soils expand into key grain-legume regions, developing salt-tolerant cultivars has become a critical priority for food security.

Among the cool-season grain legumes, faba bean (*Vicia faba* L.) occupies a position of particular nutritional and socioeconomic significance. With seed protein contents typically between 26 and 35% of dry weight and a favorable amino acid profile, it constitutes a primary protein source for rural populations across North Africa, the Near East, and South Asia^5,6^. Domesticated in the Fertile Crescent and Mediterranean during the Neolithic, the faba bean has been cultivated for over eight thousand years^7^, yet remains one of the least genomically characterized food legumes. Its large diploid genome, around 13.4 Gb, the largest among cultivated grain legumes, has historically hindered molecular breeding and dense marker development^8^. Paradoxically, the expansion of this crop’s cultivation is now focused on semi-arid and irrigation-dependent regions of Egypt, Ethiopia, and the Nile Delta areas highly vulnerable to salinization^9^. Bridging the gap between agronomic needs and genomic capabilities begins with understanding the physiological diversity within already-adapted germplasm^10^.

Salt stress affects plant health through two distinct phases that differ in timing and mechanism^11^. The first is the osmotic phase, occurring within minutes to hours of NaCl exposure, which lowers soil water potential and causes stomatal closure, thereby reducing water loss and CO_2_ intake for photosynthesis. The second, ionic phase develops over days to weeks as Na^+^ and Cl^−^ ions accumulate to toxic levels in leaf tissues actively involved in photosynthesis^11,12^. This phase hampers enzyme activity, generates reactive oxygen species, and accelerates aging^13,14^. In faba beans, these negative effects are intensified due to the plant’s particular vulnerability to the combined toxicity of Na^+^ and Cl^−^: Tavakkoli et al.^15^ showed that each ion independently harms growth, which makes faba beans especially salt-sensitive among food legumes. The most susceptible stages are germination and early seedling growth^16^, but reproductive stages, including flowering, pod formation, and seed filling, remain sensitive throughout the reproductive period. Consequently, even moderate salinity over the growing season can lead to significant economic yield losses without obvious toxicity symptoms^16^.

Plants use hierarchical responses to reduce ionic damage, including stomatal closure driven by ABA-triggered K^+^ efflux, which decreases transpiration and Na+ transport^17,18^. In quinoa, this is reinforced by lower stomatal density, which creates a structural barrier to water loss^19^. However, stomatal restriction limits CO2 availability, reducing photosynthesis and growth more than ion concentrations^20^. Cellular tolerance depends on ionic homeostasis, which maintains a proper cytosolic K^+^/Na^+^ ratio to support enzyme activity, membrane potential, and osmotic balance^21^. Na^+^ exclusion involves root Na^+^/H^+^ antiporters, tonoplast-localized NHX exchangers, and HKT transporters that recirculate Na^+^. K^+^ retention during Na+ competition involves voltage-gated channels, with genotype differences^22^. The leaf K^+^/Na^+^ ratio indicates salinity tolerance^23^. Despite understanding mechanisms, translating them into breeding is difficult owing to salt tolerance’s complex, polygenic nature, influenced by genotype, development, and stress level^24^. Screening based solely on visual traits underestimates the complexity involved and fails to effectively distinguish among various tolerance strategies. Variability in soil salinity across fields complicates testing, requiring data collection across multiple locations and seasons. Tolerance mechanisms often require significant energy expenditure, which can favor less productive genotypes. Successful phenotyping should account for the multivariate nature of the traits and remain robust across diverse environments. Currently, research on faba bean is limited; most studies examine single traits at a single NaCl level, offering only a partial understanding. Additionally, molecular insights into HKT and SOS pathways are lacking in faba bean, particularly in saline soils in Egypt. Integrating physiological and transcriptional data across different salinity levels and environments is crucial for developing scalable and effective selection strategies.

This study aimed to address that gap by hypothesizing that Egyptian faba bean cultivars, adapted to different agronomic conditions, would vary not only in salt tolerance but also in the underlying mechanisms. These cultivars are expected to exhibit distinct physiological strategies, which can be identified through a multivariate, multi-trait assessment rather than single-trait screening. To explore this, we applied three NaCl concentrations (0, 150, and 200 mM) to three cultivars (Nubaria 1, Giza 716, Sakha 5) and measured twenty traits related to morphology, physiology, and biochemistry, along with the expression of eleven salt-responsive genes involved in ion transport, osmotic regulation, antioxidant defense, transcription regulation, and wax biosynthesis. Our main goals were to: (i) examine how cultivars and treatments interact to influence trait expression under salinity; (ii) identify the mechanisms driving differences in salt tolerance using ionic, photosynthetic, root architecture, and transcriptional data; and (iii) find reliable traits for selecting salt-tolerant genotypes within this species.

## Materials and methods

### Plant material and experimental design

Three Egyptian faba bean (*Vicia faba* L.) cultivars, Nubaria 1, Giza 716, and Sakha 5, were selected on the basis of contrasting agronomic backgrounds and their cultivation in regions prone to secondary salinization. Seeds were obtained from the Grain Legumes Research Department, Field Crops Research Institute, Agricultural Research Centre, Giza, Egypt. Surface sterilization was performed by immersion in 0.5% sodium hypochlorite for five minutes, followed by three rinses in sterile distilled water. Seeds were sown at a depth of 3 cm in 5-L plastic pots filled with a sterilized sand: peat mixture (2:1, v/v), which was confirmed to have negligible initial electrical conductivity (< 0.2 dS m^− 1^). Pots were arranged in a climate-controlled greenhouse maintained at 25/18°C day/night temperatures, a 16/8 h light/dark photoperiod with supplemental illumination (photosynthetic photon flux density 400 µmol m^− 2^ s^− 1^ at canopy level), and 60–70% relative humidity throughout the experiment.

The experiment was arranged as a completely randomized design with a 3 × 3 factorial structure: three cultivars and three NaCl concentrations (0, 150, and 200 mM), with three independent pot replicates per treatment combination, giving a planned total of 27 experimental units. Salt treatment was initiated when seedlings had expanded four true leaves, approximately 21 days after sowing. To minimize osmotic shock, the target NaCl concentration was reached incrementally over three consecutive days by adding one-third of the final dose per irrigation event. Following the incremental induction period, plants were maintained under their assigned salt regime until harvest by irrigating every two to three days, or when the substrate surface appeared dry, with the respective NaCl solution (0, 150, or 200 mM) at a volume sufficient to achieve slight drainage (10–15% leachate fraction) to prevent salt accumulation above the target concentration. Free drainage was permitted through pot-base holes, and no leachate recirculation was applied. Substrate electrical conductivity (EC) was monitored weekly using a portable EC meter (HI 9813-6, Hanna Instruments) inserted directly into the growing medium at mid-pot depth; EC values remained within ± 10% of the target level throughout the experimental period (0 mM NaCl: < 0.5 dS m−¹; 150 mM NaCl: 14.2 ± 1.1 dS m−¹; 200 mM NaCl: 18.8 ± 1.3 dS m−¹). All measurements were conducted at the pod-filling stage, 60 days after sowing.

### Growth and reproductive trait measurements

At 60 days after sowing, the following parameters were recorded per plant. Plant height was measured from the soil surface to the apical meristem using a measuring rod. The numbers of primary branches, open flowers, and developed pods per plant were counted directly. Shoot and root tissues were separated at the soil surface, rinsed free of growing medium with tap water, blotted dry on absorbent paper, oven-dried at 70 °C for 72 h to constant mass, and weighed to the nearest 0.01 g to determine shoot dry weight, root dry weight, and root-to-shoot ratio.

### Root system architecture analysis

Intact root systems were separated from the growing medium by gentle washing in a water bath fitted with a 0.5 mm mesh screen to prevent fine root loss. Washed roots were suspended in a shallow water-filled tray and digitally scanned at 400 dpi using a flatbed scanner (Epson Expression 12000XL, Epson America, Long Beach, CA, USA) fitted with a transparency unit. Total root length, projected surface area, and root volume were extracted from the greyscale images using WinRHIZO Pro (version 2017a; Regent Instruments Inc., Quebec, Canada; https://regent.qc.ca/assets/winrhizo_software.html). with default threshold settings and a minimum root diameter of 0.05 mm. Scanned roots were subsequently transferred to pre-weighed paper bags, dried at 70 °C for 72 h, and weighed as described above.

### Gas exchange measurements

Gas-exchange measurements were made between 09:30 and 12:00 h solar time on the youngest fully expanded leaf of each plant using a portable open-system infrared gas analyzer (LI-6800, LI-COR Biosciences, Lincoln, NE, USA). Measurements were conducted under conditions matching the ambient greenhouse environment: a reference CO_2_ concentration of 400 µmol mol^− 1^, a photosynthetic photon flux density of 1000 µmol m^− 2^ s^− 1^ (supplied by the instrument’s LED light source), a leaf temperature of 25 ± 0.5 °C, and a vapor pressure deficit of 1.5 ± 0.2 kPa. Each measurement was initiated after a 2-min equilibration period and recorded when the coefficient of variation of net photosynthetic rate (P_n_) over a 30-s averaging window fell below 1%. The following parameters were recorded directly by the instrument: P_n_ (µmol CO_2_ m^− 2^ s^− 1^), stomatal conductance to water vapor (g_s_, mol H_2_O m^− 2^ s^− 1^), transpiration rate (T_r_, mmol H_2_O m^− 2^ s^− 1^), and intercellular CO_2_ concentration (C_i_, µmol mol^− 1^).

### Leaf mineral nutrient analysis

Immediately following gas-exchange measurements, the same youngest fully expanded leaf was excised, freeze-dried for 48 h, and ground to a fine powder in a ball mill (MM400, Retsch GmbH, Haan, Germany). Total nitrogen concentration was determined from 0.1 g of dried leaf material by micro-Kjeldahl digestion and steam distillation, using boric acid as the trapping reagent and titrating with standardized H_2_SO_4_. Phosphorus and potassium concentrations were determined from 0.2 g of dried material following wet acid digestion in a 5:1 (v/v) mixture of 65% HNO_3_ and 70% HClO_4_ on a digestion block at 180 °C until the solution cleared. Digests were diluted to 25 mL with deionized water (18.2 MΩ cm); phosphorus was quantified by the molybdate-blue colorimetric method at 882 nm, and potassium was measured by inductively coupled plasma optical emission spectrometry (ICP-OES; iCAP 7400, Thermo Fisher Scientific, Waltham, MA, USA). All nutrient concentrations are expressed on a dry weight basis (g kg^− 1^ DW).

### Epicuticular wax extraction and quantification

Epicuticular waxes were extracted from fresh leaf tissue by a brief solvent immersion method. For each sample, 1.0 g of fresh leaf tissue (upper surface area measured by scanning prior to extraction) was immersed in 20 mL of HPLC-grade chloroform in a 50-mL glass vial for exactly 30 s with gentle agitation; this brief immersion selectively removes the surface wax layer without disrupting internal lipids. The chloroform extract was transferred to a pre-weighed glass vial, and the extraction was repeated once with an additional 10 mL of chloroform; the extracts were pooled. The solvent was evaporated under a gentle stream of dry nitrogen gas at 40 °C in a water bath. The dry residue was dissolved in 100 µL of pyridine containing 2 µg of tetracosane as an internal standard, and derivatized with 100 µL of N, O-bis(trimethylsilyl)trifluoroacetamide (BSTFA; Sigma-Aldrich, St Louis, MO, USA) at 70 °C for 60 min to convert hydroxyl-bearing wax constituents (primary alcohols, fatty acids, sterols) to their trimethylsilyl ether derivatives.

Wax composition was analyzed by gas chromatography–mass spectrometry (GC-MS) on an Agilent 7890B gas chromatograph coupled to a 5977 A single-quadrupole mass-selective detector (Agilent Technologies, Santa Clara, CA, USA), fitted with a DB-5 capillary column (30 m × 0.25 mm i.d. × 0.25 μm film thickness). The programmed oven temperature was 50 °C for 2 min, ramped at 10 °C min − 1 to 200 °C, then at 5 °C min − 1 to 320 °C, and held for 15 min. Injections of 1 µL were made in splitless mode with the inlet at 300 °C; helium was the carrier gas at 1.2 mL min^− 1^ constant flow. Compound identification was based on comparison of mass spectra with the NIST 2017 library (match threshold ≥ 85%) and co-injection of authentic standards for representative odd-chain alkanes (C_27_–C_35_) and primary alcohols (C_22_–C_30_). Alkane and alcohol fractions were quantified by integration of total-ion-current chromatograms relative to the internal standard, and concentrations are expressed as µg cm^− 2^ of leaf surface area. The total wax load is reported as the sum of the alkane and alcohol fractions.

### RNA extraction, cDNA synthesis, and quantitative RT-PCR

Leaf samples for gene expression analysis were collected from the same youngest fully expanded leaf used for gas-exchange measurements, immediately snap-frozen in liquid nitrogen, and stored at − 80 °C until processing. Total RNA was extracted from 100 mg of ground frozen tissue using a modified cetyltrimethylammonium bromide (CTAB) protocol adapted for polysaccharide-rich faba bean tissue: tissue was homogenized in 900 µL of extraction buffer (2% CTAB, 2% polyvinylpyrrolidone MW 40,000, 100 mM Tris-HCl pH 8.0, 25 mM EDTA, 2 M NaCl, 2% β-mercaptoethanol, 0.05% spermidine) at 65 °C for 10 min, followed by two extractions with chloroform: isoamyl alcohol (24:1, v/v). RNA was precipitated from the aqueous phase with 0.25 volumes of 10 M LiCl at 4 °C overnight, pelleted by centrifugation (10,000 × *g*, 30 min, 4 °C), and washed twice with 70% ethanol. The pellet was resuspended in RNase-free water.

Residual genomic DNA was eliminated by on-column DNase I treatment (RNase-Free DNase Set, QIAGEN, Hilden, Germany) according to the manufacturer’s protocol. RNA integrity was assessed by denaturing agarose gel electrophoresis (1% agarose, 1× MOPS buffer); samples showing intact 28 S and 18 S rRNA bands with a 28 S/18S ratio ≥ 1.5 were used for downstream analysis. RNA concentration and purity (A_260_/A_280_ ratio 1.95–2.10) were determined using a NanoDrop One spectrophotometer (Thermo Fisher Scientific). First-strand cDNA was synthesised from 1 µg of total RNA using the RevertAid First Strand cDNA Synthesis Kit (Thermo Fisher Scientific) with oligo(dT)_18_ primers, following the manufacturer’s thermocycling programme (42 °C for 60 min, 70 °C for 5 min).

Quantitative real-time PCR (qRT-PCR) was performed on a LightCycler^®^ 480 System (Roche Life Science, Penzberg, Germany) using SYBR Green I Master Mix (Roche) in a final reaction volume of 20 µL, containing 4 µL of 1:10 diluted cDNA, 10 µL of 2× SYBR Green Master Mix, and 0.4 µM of each primer. Thermal cycling conditions were: 95 °C for 5 min (initial denaturation); 45 cycles of 95 °C for 10 s, 60 °C for 20 s, and 72 °C for 20 s; followed by a melt-curve analysis from 65 to 97 °C in 0.1 °C increments to confirm amplicon specificity. All reactions were performed in triplicate, and no-template and no-reverse-transcriptase controls were included on each plate. Primer sequences, amplicon lengths, melting temperatures, and GenBank accession numbers for the eleven target genes (*SOS1*, *HKT1*, *PIP2*, *P5CS*, *P5CR*, *CAT*, *SOD*, *APX*, *DREB2*, *LEA*, *CER1*) and two reference genes (*ACT* encoding actin; *UBQ* encoding ubiquitin) are provided in Table S1. All primers used in this study were designed directly from confirmed *Vicia faba*-specific mRNA sequences deposited in GenBank (accession numbers listed in Table S1), with the exception of *HKT1* and *DREB2*, for which primers were designed from homologous sequences in *Pisum sativum* (sequence identity ≥ 91% at the nucleotide level across the amplicon region, verified by BLASTn against the *V. faba* transcriptome). Amplicon specificity in faba bean was confirmed by single-peak melt curves for all 11 target genes and by gel electrophoresis of representative PCR products, which showed single bands of the expected size (Table S1). Primer efficiency was validated for each gene by a five-point 10-fold serial dilution of pooled cDNA; all efficiencies were within 95–105% (R^2^ ≥ 0.99). Reference gene stability across all cultivar–treatment combinations was confirmed using geNorm (version 3.5), with both *ACT* and *UBQ* returning stability values (M) below 0.5. Relative expression levels were calculated by the 2^−ΔΔCT^ method^25^, normalized to the geometric mean of the two reference genes, with the control treatment of each respective cultivar set as the calibrator. All qRT-PCR data are reported as fold-change relative to this within-cultivar control baseline.

### Statistical analysis

All trait data were subjected to two-way analysis of variance (ANOVA) with cultivar (C) and NaCl treatment (T) as fixed factors and their interaction (C×T), using the aov() function in R version 4.2.1 (R Core Team, 2022). The assumptions of normality and homogeneity of variance were evaluated prior to ANOVA using the Shapiro–Wilk test and Levene’s test, respectively; no transformations were required for any variable. Where ANOVA indicated significant effects (*P* < 0.05), means were compared by Tukey’s honest significant difference (HSD) post-hoc test using the TukeyHSD() function, and compact letter displays were generated with the multcompView package.

Pearson correlation coefficients among all measured traits were computed separately for each NaCl treatment level using the cor() function, and significance was assessed by a two-tailed t-test with Bonferroni correction for multiple comparisons. Correlation matrices were visualised as heatmaps using the corrplot package. Principal component analysis (PCA) was performed on the full standardized dataset (mean-centred, unit-variance scaled) using the prcomp() function; biplots were produced with the factoextra package, with 95% concentration ellipses drawn by cultivar. Expression data from qRT-PCR were analyzed separately using a linear mixed-effects model with cultivar and NaCl treatment as fixed effects and biological replicate as a random effect, implemented via the lme4 package; pairwise contrasts among treatment means within each gene were evaluated by Tukey’s HSD at α = 0.05. All figures were produced in ggplot2 (version 3.4.0). Data are presented as means ± standard error of the mean (SEM) unless otherwise stated.

## Results

### Analysis of variance for cultivar and salt stress effects

Two-way analysis of variance partitioned the total variation in each trait among three sources: cultivar (C), NaCl treatment (T), and their interaction (C×T) (Table 1). The results differed substantially among trait categories. For traits related to plant architecture and biomass allocation, branch number, root dry weight, root-to-shoot ratio, and root volume, the cultivar term accounted for the largest mean squares, with highly significant effects (*P* < 0.001) and non-significant treatment and interaction terms. These traits, therefore, varied among genotypes regardless of NaCl level. Leaf nitrogen concentration followed the same pattern, with a significant cultivar effect and no significant treatment or interaction effects. Gas-exchange traits, net photosynthesis, stomatal conductance, transpiration rate, and intercellular CO₂ concentration, showed the largest mean squares under the treatment term, indicating that NaCl level was the dominant source of variation for these parameters. Significant C×T interactions were detected for net photosynthesis, stomatal conductance, and intercellular CO₂, indicating that the magnitude of the treatment effect on these traits differed among cultivars. Epicuticular wax fractions, alkane, alcohol, and total wax were similarly dominated by the treatment term, with significant C×T interactions for all three components, confirming that the wax response to salinity was not uniform across genotypes. Root length showed significant effects of both cultivar and treatment, and the C×T interaction was also highly significant, indicating that the direction and magnitude of the root length response to NaCl differed among genotypes. Leaf potassium concentration showed significant effects of cultivar, treatment, and their interaction, making it one of a small number of traits for which all three sources of variation were statistically distinguishable. Reproductive traits, flower number, and pod number showed significant effects of both cultivar and treatment, with a significant C×T interaction for flower number. Phosphorus concentration was significantly affected by cultivar and treatment, but there was no significant interaction between them. Taken together, the ANOVA results show that the measured traits fell into three broad patterns: those determined primarily by cultivar identity and unaffected by treatment; those driven primarily by NaCl level and similarly affected across cultivars; and a subset root length, leaf K⁺, gas-exchange parameters, and wax fractions for which the response to treatment differed significantly among genotypes.

**Table 1:** Mean square values from two-way ANOVA of all measured traits partitioned by cultivar (C), NaCl treatment (T), and their interaction (C×T).

Source	Plant Height (cm)	Branches/plant	Flowers/plant	Pods/plant	Shoot weight (g/plant)	Root weight (g/plant)	Root/Shoot ratio	Root length (cm)	Root area (cm²)	Root volume (cm³)
Cultivar	963.65***	16.05***	597.34***	7.607***	0.16*	1.11***	0.091***	543.62***	178.01***	388.44***
Treatment	528.44**	0.06	22.07***	0.74**	3.89***	0.18*	0.0099	502.57***	180.28	21.252
Cultivar×Treatment	25.93	0.12	5.71*	0.24	0.11	0.07	0.003	58.22***	45.67	23.78
Residuals	57.94	0.51	1.48	0.08	0.041	0.05	0.003	22.35	29.36	18.128
**Source**	**N content (g/kg)**	**P content (g/kg)**	**K content (g/kg)**	**Net photosynthesis**	**Stomatal conductance**	**Transpiration**	**Intercellular CO₂**	**Alkane**	**Alcohol**	**Total wax**
Cultivar	43.53***	0.00012***	2.55***	4.97***	0.0009***	0.049*	511.06***	0.002	0.3228	9.312*
Treatment	4.76	5.01**	1.83***	55.22***	0.013***	0.34***	4760.58***	1.202***	17.47***	45.01***
Cultivar×Treatment	2.24	6.87	1.68***	1.48***	0.0002***	0.02	620.96***	0.690***	0.494**	9.08**
Residuals	2.38	5.22	0.13	0.06	5.92	0.009	27.88	0.041	0.096	1.73

Significance codes: *** P < 0.001; ** P < 0.01; * P < 0.05; ns not significant.

### Effects of salt stress on growth and reproductive parameters

**Fig. 1:**
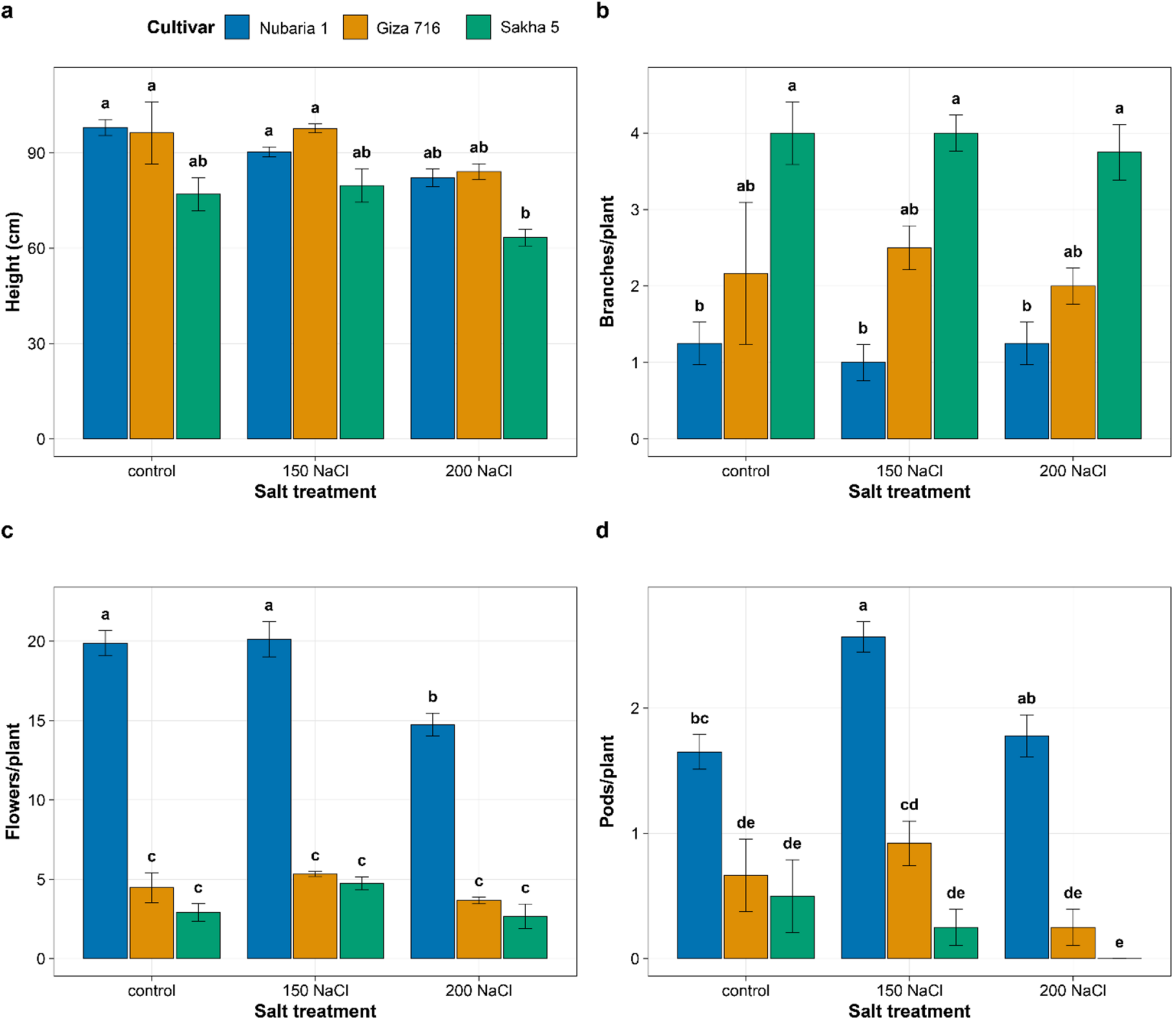
Effects of NaCl treatment on (**a**) plant height, (**b**) branch number, (**c**) flower number, and (**d**) pod number per plant in three Vicia faba cultivars. Bars represent means ± SE. Different letters above bars within each panel indicate significant differences (Tukey’s HSD, *P* < 0.05).

Salt stress progressively suppressed vegetative growth across all three cultivars, although the magnitude and pattern of reduction differed markedly among genotypes (Fig. 1a-d). Under control conditions, Giza 716 was the tallest cultivar (mean 105.9 cm), followed by Nubaria 1 (98.0 cm) and Sakha 5 (77.0 cm). Increasing NaCl concentration reduced plant height in all three genotypes; at 200 mM NaCl, heights declined to 84.1, 82.2, and 63.3 cm in G 716, Nubaria 1, and SAKHA 5, respectively, representing reductions of 21%, 16%, and 18% from their respective controls. Sakha 5 thus experienced the greatest absolute height loss under severe salinity conditions (Fig. 1a).

Branch number did not change significantly with NaCl treatment or with the cultivar-by-treatment interaction, consistent with the ANOVA results in Table 1 (Fig. 1b). Sakha 5 produced the most branches per plant across all conditions (mean 4.0 at control, 3.75 at 200 mM NaCl), while Nubaria 1 and Giza 716 had lower and comparably stable branch counts throughout (1.25 and 1.25–2.5 per plant, respectively).

Flower number differed significantly among cultivars and was reduced by NaCl treatment, with a significant C×T interaction (Table 1; Fig. 1c). Under control conditions, Nubaria 1 produced the most flowers per plant (mean 19.9), followed by Giza 716 (5.3) and Sakha 5 (2.9). At 200 mM NaCl, flower counts declined to 14.8, 3.7, and 2.7 per plant in Nubaria 1, Giza 716, and Sakha 5, respectively, representing reductions of 30%, 30%, and 8% from their respective controls. The smaller proportional decline in Sakha 5 at 200 mM NaCl reflects its low baseline value under control conditions rather than a difference in absolute flower loss (Fig. 1c).

Pod number also differed among cultivars and declined with increasing NaCl, with a significant treatment effect (Table 1; Fig. 1d). Under control conditions, Nubaria 1 set the most pods per plant (1.65), followed by Giza 716 (0.75) and Sakha 5 (0.50). At 150 mM NaCl, pod number increased in Nubaria 1 (to 2.57) while remaining comparably low in Giza 716 (0.75) and Sakha 5 (0.50). At 200 mM NaCl, pod number declined in all cultivars: Nubaria 1 retained 1.78 pods per plant, Giza 716 fell to 0.25, and Sakha 5 produced zero pods.

### Biomass accumulation and partitioning under salt stress

**Fig. 2:**
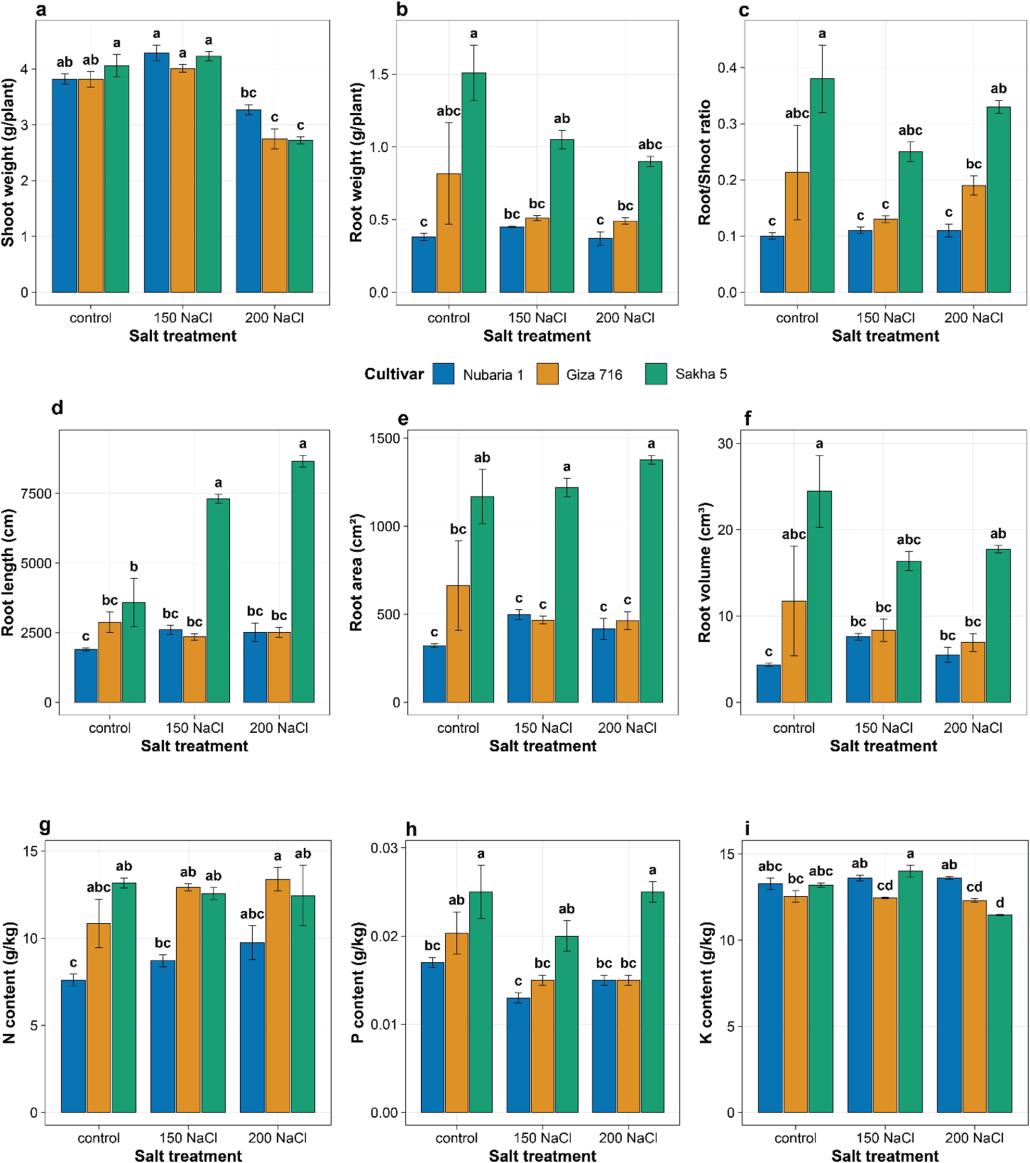
Effects of NaCl treatment on (**a**) shoot dry weight, (**b**) root dry weight, (**c**) root-to-shoot, (**d**) total root length, (**e**) root surface area, and (**f**) root volume, (**g**) nitrogen, (**h**) phosphorus, and (**i**) potassium in three Vicia faba cultivars. Bars represent means ± SE. Different letters indicate significant differences (Tukey’s HSD, *P* < 0.05).

Shoot dry weight differed among cultivars and declined with increasing NaCl concentration, though the magnitude of decline varied among genotypes (Fig. 2a). Under control conditions, Sakha 5 produced the highest shoot biomass (4.06 g plant⁻¹), followed by Nubaria 1 (3.82 g plant⁻¹) and Giza 716 (3.69 g plant⁻¹). At 200 mM NaCl, shoot weight declined to 3.27, 2.75, and 2.72 g plant⁻¹ in Nubaria 1, Giza 716, and Sakha 5, respectively, representing reductions of 14%, 25%, and 33% from their respective controls. Nubaria 1 therefore showed the smallest proportional decline in shoot dry weight between control and 200 mM NaCl. Root dry weight differed substantially among cultivars across all treatment levels (Fig. 2b). Under control conditions, Sakha 5 produced the heaviest root system (1.51 g plant⁻¹), approximately 3.2-fold greater than Giza 716 (0.47 g plant⁻¹) and 4.0-fold greater than Nubaria 1 (0.38 g plant⁻¹). At 200 mM NaCl, root dry weight declined in Sakha 5 (to 0.90 g plant⁻¹, a 40% reduction) and remained essentially unchanged in Nubaria 1 (0.37 g plant⁻¹, a 3% reduction), while Giza 716 showed a slight increase (to 0.49 g plant⁻¹). The root-to-shoot ratio differed markedly among cultivars, being higher in Sakha 5 than in either Giza 716 or Nubaria 1 across all treatment levels (Fig. 2c). Under control conditions, the ratio was 0.38 in Sakha 5, 0.13 in Giza 716, and 0.10 in Nubaria 1. At 200 mM NaCl, the ratio increased in Giza 716 (to 0.19) and remained similar in Sakha 5 (to 0.33) and in Nubaria 1 (to 0.11). The higher root-to-shoot ratio in Sakha 5 was present under control conditions and was maintained, rather than induced, across NaCl treatments.

### Root system architecture responses to salinity

Root architectural responses to salinity differed markedly among the three cultivars in both magnitude and direction (Fig. 2d–f). Under control conditions, Sakha 5 possessed the most extensive root system, with total root length (3,577 cm), root surface area (1,169 cm²), and root volume (24.45 cm³) each approximately 2.8–5.6-fold greater than those of Nubaria 1 (1,900 cm; 322 cm²; 4.35 cm³) and Giza 716 (2,529 cm; 409 cm²; 5.37 cm³). Root length in Sakha 5 increased substantially with increasing NaCl concentration: from 3,577 cm at control to 7,315 cm at 150 mM NaCl (+ 104%) and to 8,664 cm at 200 mM NaCl (+ 142). Root length in Nubaria 1 increased modestly at 150 mM NaCl (to 2,605 cm, + 37%) and was essentially unchanged at 200 mM NaCl (2,519 cm, + 33%). Root length in Giza 716 remained stable across all treatment levels (2,350–2,514 cm), with no significant effect of NaCl concentration. The cultivar-by-treatment interaction for root length was highly significant (*P* < 0.001; Table 1), confirming that the direction and magnitude of the root length response to salinity differed among genotypes. Root surface area and root volume in Sakha 5 also remained substantially elevated under stress (1,220–1,377 cm² and 16.4–17.7 cm³, respectively), whereas these parameters showed comparatively modest and non-significant changes in Nubaria 1 and Giza 716 across treatments. The two-way ANOVA indicated that root surface area and root volume were not significantly affected by treatment or by the cultivar-by-treatment interaction (Table 1), whereas root length showed a significant interaction. This suggests that the stress-induced changes in Sakha 5 were primarily expressed as elongation rather than an increase in root diameter or branching density.

### Nutrient accumulation and homeostasis under salt stress

Leaf nitrogen concentration differed significantly among cultivars but was not significantly affected by NaCl treatment or the cultivar-by-treatment interaction (Table 1; Fig. 2g). Under control conditions, Sakha 5 had the highest leaf N (13.2 g kg⁻¹), followed by Giza 716 (9.7 g kg⁻¹) and Nubaria 1 (7.6 g kg⁻¹). Values remained within a comparably narrow range across all NaCl levels in each cultivar (9.5–15.4 g kg⁻¹ across all cultivar-treatment combinations).

Leaf phosphorus concentration was significantly affected by both cultivar and NaCl treatment, with no significant interaction (Table 1; Fig. 2h). Under control conditions, Sakha 5 had the highest leaf P (0.025 g kg⁻¹), followed by Giza 716 (0.018 g kg⁻¹) and Nubaria 1 (0.017 g kg⁻¹). All three cultivars showed declines in leaf P with increasing NaCl, with values falling to approximately 0.013–0.025 g kg⁻¹ at 200 mM NaCl. The absence of a significant cultivar-by-treatment interaction indicates that the direction and magnitude of the decline were similar across genotypes.

Leaf potassium concentration was significantly affected by cultivar, NaCl treatment, and their interaction (*P* < 0.001 for all three terms; Table 1; Fig. 2i). Under control conditions, Nubaria 1 had the highest leaf K (13.3 g kg⁻¹), with Sakha 5 (13.2 g kg⁻¹) and Giza 716 (12.2 g kg⁻¹) slightly lower. At 200 mM NaCl, the three cultivars diverged: Nubaria 1 showed no decline (13.6 g kg⁻¹), Giza 716 declined modestly (to 12.3 g kg⁻¹), and Sakha 5 showed the largest reduction (to 11.5 g kg⁻¹). Leaf K therefore followed different trajectories across NaCl levels in the three cultivars, which accounts for the significant interaction term.

### Gas exchange parameters and photosynthetic responses

**Fig. 3:**
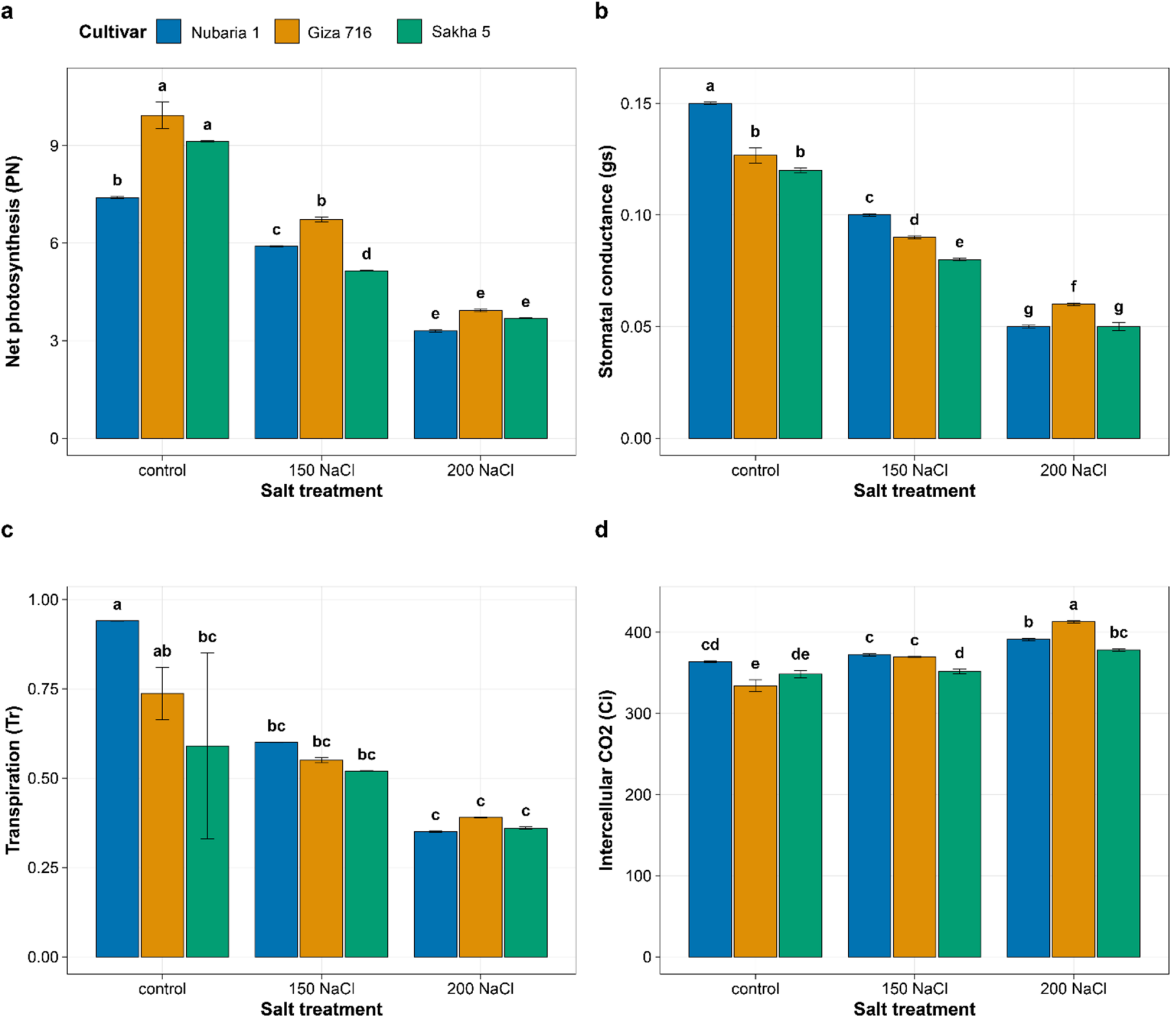
Effects of NaCl treatment on (**a**) net photosynthetic rate (Pₙ), (**b**) stomatal conductance (gₛ), (**c**) transpiration rate (T_r_), and (**d**) intercellular CO₂ concentration (C_i_) in three Vicia faba cultivars. Measurements were made on the youngest fully expanded leaf using a portable LI-6800 gas analyser. Bars represent means ± SE. Different letters indicate significant differences (Tukey’s HSD, *P* < 0.05).

Gas exchange was significantly affected by NaCl treatment, with significant cultivar-by-treatment interactions for net photosynthesis, stomatal conductance, and intercellular CO₂ concentration (Table 1; Fig. 3). Under control conditions, Giza 716 had the highest net photosynthetic rate (Pₙ = 10.33 µmol CO₂ m⁻² s⁻¹), followed by Sakha 5 (9.13 µmol CO₂ m⁻² s⁻¹) and Nubaria 1 (7.40 µmol CO₂ m⁻² s⁻¹) (Fig. 3a). At 200 mM NaCl, Pₙ declined to 3.93, 3.69, and 3.30 µmol CO₂ m⁻² s⁻¹ in Giza 716, Sakha 5, and Nubaria 1, respectively, representing reductions of 62%, 60%, and 55% from their respective controls. Stomatal conductance (gₛ) declined in all cultivars with increasing NaCl (Fig. 3b). At 200 mM NaCl, reductions from control were 67%, 54%, and 58% in Nubaria 1 (0.150 → 0.050 mol H₂O m⁻² s⁻¹), Giza 716 (0.130 → 0.060 mol H₂O m⁻² s⁻¹), and Sakha 5 (0.120 → 0.050 mol H₂O m⁻² s⁻¹), respectively. Transpiration rate (T_r_) followed a similar pattern of decline across cultivars and treatment levels (control: 0.94, 0.81, and 0.59 mmol H₂O m⁻² s⁻¹ in Nubaria 1, Giza 716, and Sakha 5; 200 mM NaCl: 0.35, 0.39, and 0.36 mmol H₂O m⁻² s⁻¹) (Fig. 3c). Intercellular CO₂ concentration (C_i_) increased in all cultivars with increasing NaCl (Fig. 3d). Under control conditions, Giza 716 had the lowest C_i_ (327 µmol mol⁻¹), while Nubaria 1 (364 µmol mol⁻¹) and Sakha 5 (348 µmol mol⁻¹) were higher. At 200 mM NaCl, C_i_ increased to 391, 413, and 378 µmol mol⁻¹ in Nubaria 1, Giza 716, and Sakha 5, respectively. The decline in Pₙ therefore, occurred simultaneously with a rise in C_i_ in all three cultivars at both stress levels.

### Epicuticular wax accumulation as a stress response

**Fig. 4:**
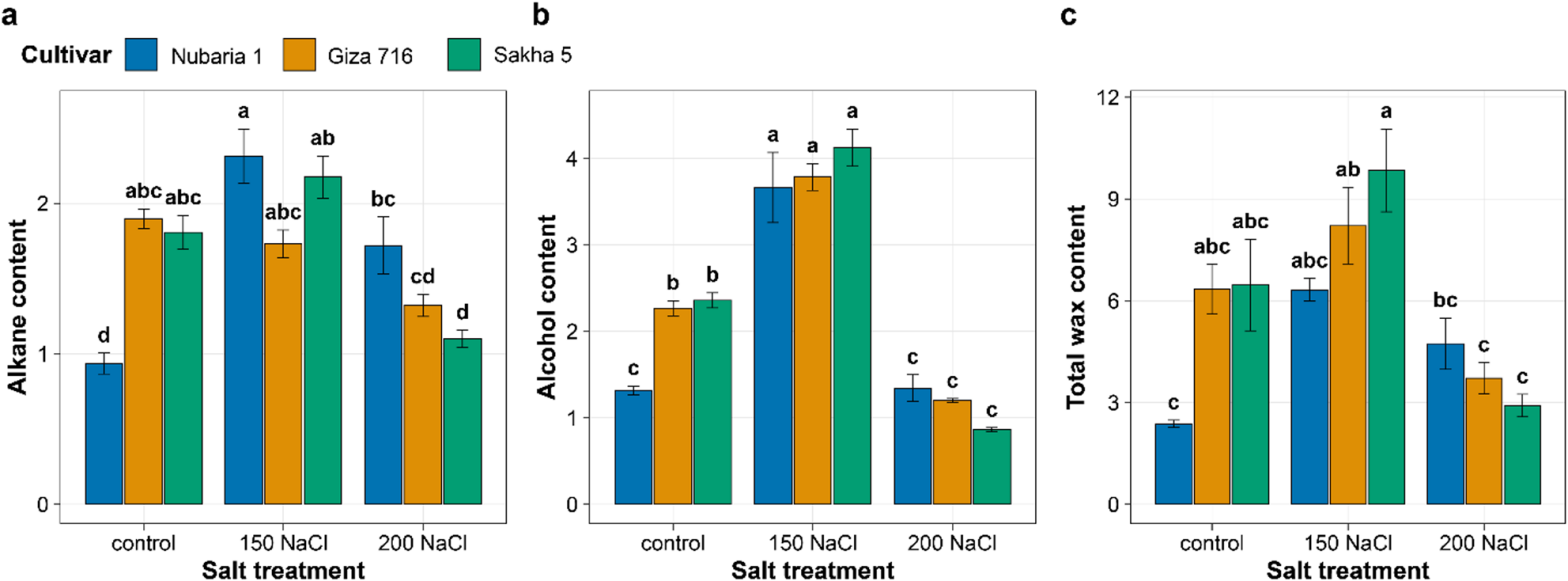
Effects of NaCl treatment on leaf epicuticular wax components: (**a**) alkane content, (**b**) alcohol content, and (**c**) total wax load in three *Vicia faba* cultivars. Wax was extracted from fresh leaf tissue by chloroform immersion and quantified by GC-MS. Bars represent means ± SE. Different letters indicate significant differences (Tukey’s HSD, *P* < 0.05).

Epicuticular wax composition and total load changed with NaCl treatment in all three cultivars, with significant cultivar-by-treatment interactions for all three wax components (Table 1; Fig. 4). Under control conditions, Sakha 5 and Giza 716 had comparably higher total wax loads (6.46 and 6.27 µg cm⁻², respectively) than Nubaria 1 (2.37 µg cm⁻²) (Fig. 4a). At 150 mM NaCl, total wax increased in all three cultivars, reaching 9.85, 8.21, and 6.32 µg cm⁻² in Sakha 5, Giza 716, and Nubaria 1, respectively. At 200 mM NaCl, total wax declined from its 150 mM peak in Sakha 5 (to 2.91 µg cm⁻²) and Giza 716 (to 3.72 µg cm⁻²), while Nubaria 1 showed a smaller decline from its 150 mM value (to 4.74 µg cm⁻²). Nubaria 1, therefore, had the highest total wax load of the three cultivars at 200 mM NaCl, despite having the lowest under control conditions. Alkane content increased at 150 mM NaCl in Nubaria 1 (0.94 → 2.31 µg cm⁻², + 146%) and Sakha 5 (1.81 → 2.17 µg cm⁻², + 20%), while it declined in Giza 716 (1.94 → 1.73 µg cm⁻²) (Fig. 4b). At 200 mM NaCl, alkane content decreased across all three cultivars relative to 150 mM NaCl, with Nubaria 1 retaining the highest alkane fraction (1.72 µg cm⁻²). Wax alcohol content increased at 150 mM NaCl in all cultivars, with the largest absolute increase in Sakha 5 (2.36 → 4.12 µg cm⁻², + 75%) (Fig. 4c). At 200 mM NaCl, alcohol content declined from its 150 mM peak in Sakha 5 and Giza 716, while remaining higher than control levels in all three cultivars.

### Trait correlations under different salt stress levels

**Fig. 5:**
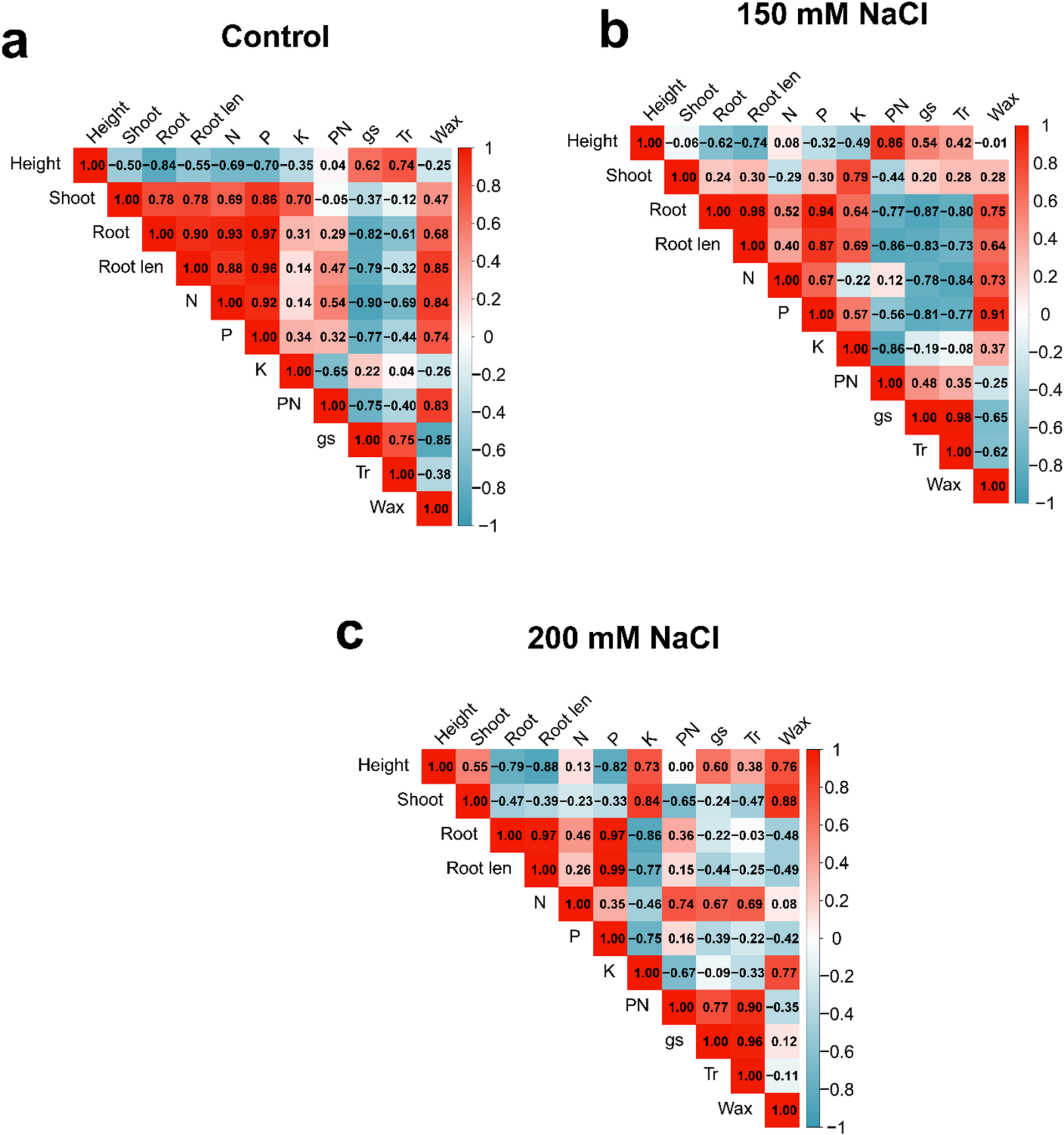
Pearson correlation matrices for all 20 measured traits under (**a**) control, (**b**) 150 mM NaCl, and (**c**) 200 mM NaCl conditions. Colour scale indicates correlation coefficient (r) from − 1 (red) to + 1 (blue). Significant correlations (*P* < 0.05) are indicated by asterisks.

Pearson correlation analysis showed that the structure of trait associations differed among the three salinity levels, with the number and identity of significant correlations changing as NaCl concentration increased (Fig. 5). Under control conditions, plant height was negatively correlated with root dry weight (*r* = − 0.84) and root length (*r* = − 0.55) (Fig. 5a). Root morphological traits — root length, root surface area, and root volume were strongly intercorrelated (*r* = 0.78–0.96). Leaf nitrogen and phosphorus co-varied positively (*r* = 0.92), and both were positively associated with root morphological traits. Within the gas-exchange measurements, Pₙ was positively correlated with gₛ (*r* = 0.62) and T_r_ (*r* = 0.74) and negatively correlated with C_i_ (*r* = − 0.90). Total wax load was positively associated with root biomass and nutrient traits. Under moderate salinity (150 mM NaCl), several correlations changed in magnitude or direction relative to the control (Fig. 5b). The negative association between height and root architecture traits persisted (*r* = − 0.62 to − 0.74), while the correlation between height and shoot weight was near zero (*r* = − 0.06). Root traits remained strongly intercorrelated (*r* > 0.87). The positive correlation between Pₙ and gₛ weakened (*r* = 0.54 vs. 0.62 at control), and the negative correlation between Pₙ and C_i_ declined substantially (*r* = 0.12 vs. −0.90 at control). Total wax correlated positively with root length (*r* = 0.85) and nutrient traits (*r* = 0.73–0.91) at this treatment level. Under severe salinity (200 mM NaCl), root architectural traits were more tightly intercorrelated than at any other treatment level (*r* > 0.97) and were positively associated with phosphorus content (*r* = 0.97–0.99) (Fig. 5c). Leaf potassium showed negative correlations with root morphological traits (*r* = − 0.75 to − 0.86). Stomatal conductance and transpiration remained strongly coupled (*r* = 0.96) and were both positively correlated with Pₙ (*r* = 0.77–0.90). Total wax was positively correlated with plant height (*r* = 0.76) and shoot dry weight (*r* = 0.88), and weakly or negatively correlated with root traits.

### Principal component analysis of cultivar responses

**Fig. 6:**
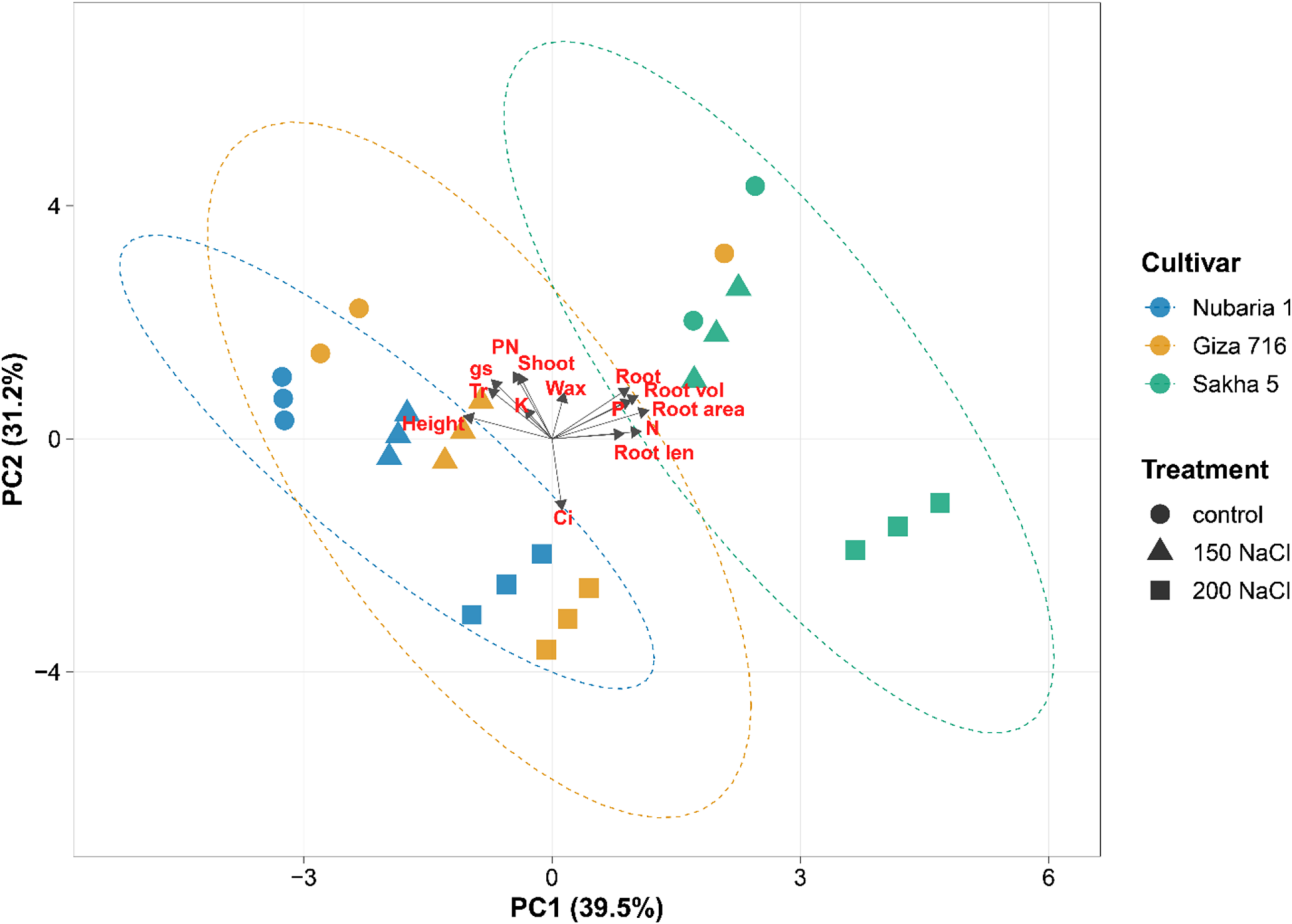
Principal component analysis biplot of all 26 observations across the three cultivars and three NaCl treatments. Data were standardized (mean = 0, SD = 1) prior to analysis. Points are colour-coded by cultivar and shaped by treatment; 95% concentration ellipses are drawn by cultivar. Trait loading vectors are shown for the 10 traits contributing most to the first two principal components.

Principal component analysis of the full standardized dataset produced two components that together explained 70.7% of total variance (PC1: 39.5%; PC2: 31.2%) (Fig. 6). Cultivar samples were separated along both components, with Sakha 5, Nubaria 1, and Giza 716 occupying distinct regions of the biplot that remained consistent across treatment levels. On PC1, Sakha 5 samples had the highest positive scores, associated with large positive loadings for root length, root surface area, root volume, and root dry weight. Nubaria 1 samples had the most negative PC1 scores, associated with positive loadings for flower number and pod number. Giza 716 samples were intermediate on PC1. On PC2, control samples scored most positively, 150 mM NaCl samples were intermediate, and 200 mM NaCl samples scored most negatively. Shoot dry weight, total wax load, and plant height had positive loadings on PC2, while intercellular CO₂ had a strong negative loading. Net photosynthesis and stomatal conductance also loaded positively on PC2. Within each cultivar, the 95% concentration ellipses were elongated along PC2, indicating that within-cultivar variation was associated primarily with treatment level. The ellipses for the three cultivars remained separated along PC1 across all treatment levels, with no overlap among cultivar groupings. The ten traits with the largest loadings on PC1 and PC2 are indicated by vectors in Fig. 6.

### Differential expression of salt-responsive genes

**Fig. 7:**
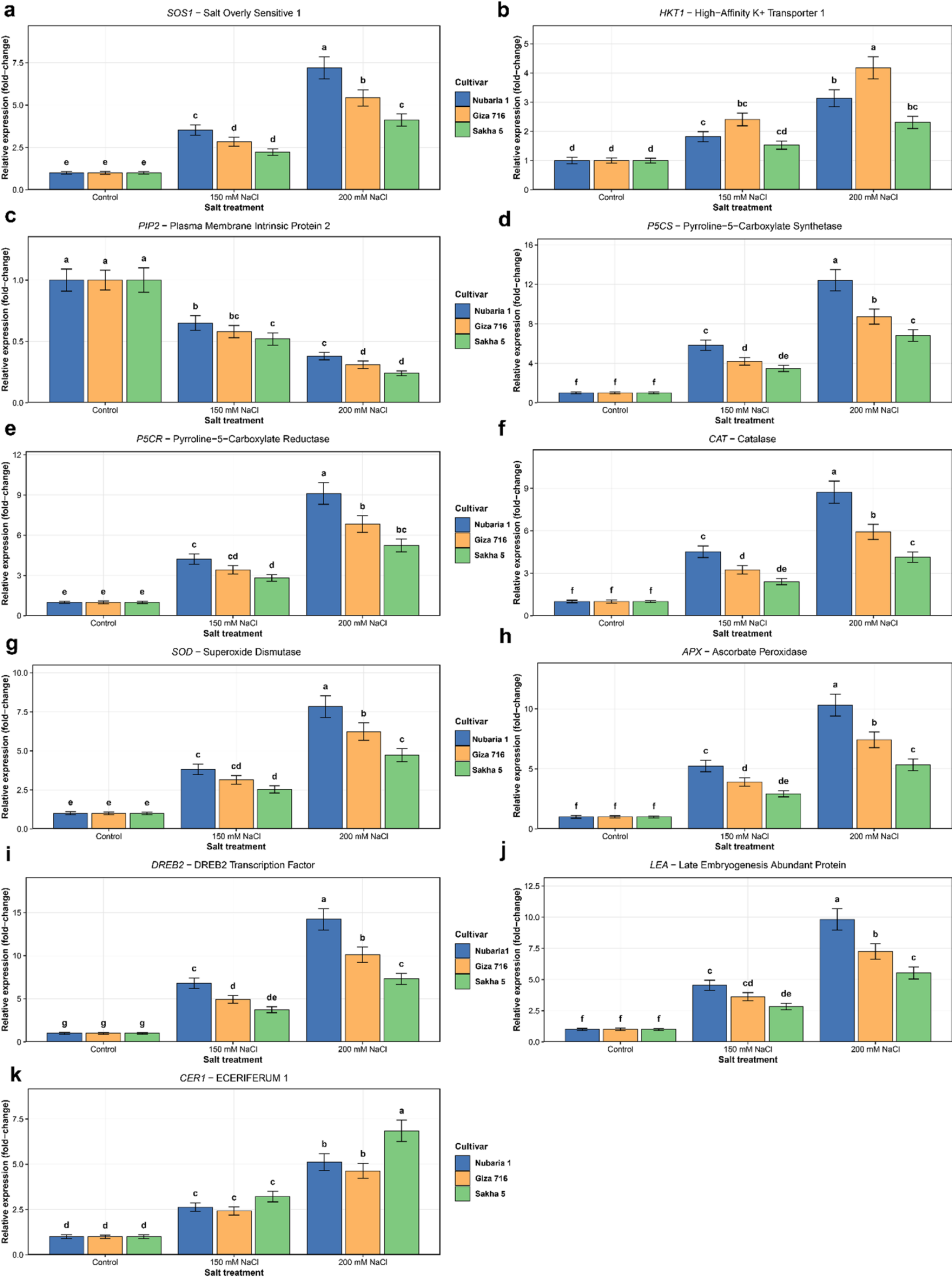
Relative expression (fold-change relative to the control of each cultivar) of eleven salt-responsive genes in three Vicia faba cultivars under control, 150 mM NaCl, and 200 mM NaCl conditions. Genes are grouped by functional category: (**a**) ion transport (SOS1, HKT1, PIP2); (**b**) osmotic adjustment (P5CS, P5CR); (**c**) antioxidant defence (CAT, SOD, APX); (**d)** transcriptional regulation and late stress response (DREB2, LEA); (**e**) wax biosynthesis (CER1). Bars represent means ± SE of three biological replicates, each measured in technical triplicate. Reference genes: ACT (actin) and UBQ (ubiquitin). Different letters within each panel indicate significant differences (Tukey’s HSD, *P* < 0.05).

Quantitative RT-PCR of eleven genes spanning ion transport, osmotic adjustment, antioxidant defense, transcriptional regulation, and wax biosynthesis revealed consistent, cultivar-stratified differences in induction magnitude across NaCl treatments (Fig. 7a–k). Across all gene categories, Sakha 5 showed the highest fold-induction, Giza 716 was intermediate, and Nubaria 1 showed the lowest induction. It should be noted that these fold-changes are expressed relative to each cultivar’s own within-treatment control baseline and therefore do not reflect absolute transcript levels, which may differ among genotypes independently of stress induction. *SOS1* expression was progressively upregulated by NaCl across all cultivars, reaching approximately 6–7-fold in Sakha 5 at 150–200 mM NaCl, compared with less than 2-fold in Nubaria 1 (Fig. 7a). *HKT1* induction was highest in Sakha 5 at 150 mM NaCl (4.5-fold) and declined at 200 mM NaCl, while Nubaria 1 showed less than 2-fold change across all treatments (Fig. 7b). *PIP2* showed the opposite pattern: expression was relatively maintained in Nubaria 1 and declined progressively in Giza 716 and most strongly in Sakha 5, which reached approximately 0.2-fold at 200 mM NaCl (Fig. 7c). *P5CS* and *P5CR*, which encode committed steps of proline biosynthesis, were most strongly upregulated in Sakha 5 (11–13-fold and 8–10-fold, respectively, at 150–200 mM NaCl), intermediate in Giza 716 (6–7-fold for *P5CS*; 4–5-fold for *P5CR*), and minimally induced in Nubaria 1 (less than 1.5-fold across all treatments) (Fig. 7d–e). *CAT*, *SOD*, and *APX* followed the same cultivar ranking across all treatments (Sakha 5 > > Giza 716 > > Nubaria 1) (Fig. 7f–h). In Sakha 5, *CAT* and *APX* reached approximately 7–8-fold induction at 150 mM NaCl, with *APX* remaining at 7-fold at 200 mM NaCl. *SOD* was similarly elevated in Sakha 5 (7–8-fold). Nubaria 1 showed less than a 2-fold change for all three genes across all treatments.

*DREB2* showed the largest fold-induction of any gene tested: 12–13-fold in Sakha 5 at 150 mM NaCl, declining to 10-fold at 200 mM NaCl; 4–5-fold in Giza 716; and below 1-fold in Nubaria 1 throughout (Fig. 7i). *LEA* followed a similar pattern, with Sakha 5 reaching 10–12-fold, Giza 716 4–5-fold, and Nubaria 1 remaining near baseline (Fig. 7j). The co-induction of *DREB2* with the osmotic adjustment, antioxidant, and *LEA* genes in Sakha 5 is consistent with a coordinated transcriptional response to ionic stress, though whether *DREB2* acts as a regulator of these co-induced genes in this context was not examined. Wax biosynthesis gene. *CER1* was progressively induced with increasing NaCl in Sakha 5 (approximately 4-fold at 150 mM NaCl, rising to 6-fold at 200 mM NaCl) and in Giza 716 (2.5–3-fold), while Nubaria 1 showed only marginal induction (Fig. 7k). Notably, *CER1* induction in Sakha 5 continued to increase at 200 mM NaCl despite a decline in total wax load at that treatment level, demonstrating that transcript abundance alone did not predict wax accumulation. This disconnect illustrates a general caution for interpreting the fold-change data across this dataset: transcriptional induction is not a reliable proxy for metabolic output, and biochemical or post-transcriptional constraints may uncouple gene expression from the traits being measured. Taken together, the expression data show that Sakha 5 mounted the broadest and largest-magnitude transcriptional response across all gene categories, while Nubaria 1 showed the smallest induction across all pathways. Giza 716 was consistently intermediate. These differences in transcriptional response pattern coincide with the cultivar differences in growth, ion status, and gas exchange described above, but causal relationships between specific transcriptional changes and phenotypic outcomes cannot be established from these data.

## Discussion

The three Egyptian faba bean cultivars examined here differed not only in the degree of growth suppression under NaCl stress but also in the pattern of their physiological and transcriptional responses, as indicated by the significant cultivar-by-treatment interactions detected for root length, leaf K⁺, gas-exchange parameters, and wax fractions. Nubaria 1 showed the smallest reductions in shoot biomass, leaf K⁺, net photosynthesis, and pod set under severe salinity, and exhibited the lowest transcriptional induction across all 11 genes. Sakha 5 showed the largest transcriptional induction in all gene categories and the greatest root elongation under stress, yet suffered complete pod failure at 200 mM NaCl. Giza 716 was intermediate in both respects, with the highest baseline net photosynthetic rate under control conditions. These observations are broadly consistent with the framework of Munns and Tester^26^, which distinguishes between genotypes that differ in ion exclusion, tissue tolerance, and osmotic adjustment, though the specific mechanisms operating in each cultivar here require further investigation to confirm.

Nubaria 1 maintained leaf K⁺ concentration at 13.3–13.6 g kg⁻¹ across all NaCl levels, while Sakha 5 declined to 11.5 g kg⁻¹ at 200 mM NaCl, and Giza 716 showed a modest intermediate decline. This cultivar difference in leaf K⁺ retention under stress is consistent with differences in ion selectivity at the root–soil interface, possibly involving HAK/KT family transporters or post-translationally regulated voltage-gated channels^27–29^. However, because leaf and root Na⁺ and Cl⁻ concentrations were not measured in this study, it is not possible to determine whether the K⁺ differences reflect differential Na⁺ exclusion, differential K⁺ retention under Na⁺ competition, or both. The low *HKT1* and *SOS1* induction in Nubaria 1, relative to Sakha 5, is consistent with a smaller ionic perturbation in that cultivar, but cannot be interpreted mechanistically without ion accumulation data. Direct measurement of tissue ion composition across cultivars and salinity levels is, therefore, necessary before any conclusion about the ion-transport mechanisms responsible for K⁺ retention in Nubaria 1 can be drawn.

The progressive elongation of the Sakha 5 root system from 3,577 cm under control to 8,664 cm at 200 mM NaCl (+ 142%) was the most striking morphological finding of this study and distinguishes this cultivar from both Nubaria 1 and Giza 716, which showed modest or no increases in root length. Salt-stimulated root elongation has been reported in other species and has been associated with auxin redistribution affecting polar PIN-mediated transport^30,31^. The co-induction of *P5CS* and *P5CR* in Sakha 5 under the same conditions is consistent with increased proline availability in growing root cells, which could contribute to osmotic adjustment supporting cell elongation^32,33^. Whether the root elongation observed here represents an adaptive response that improves resource access or a stress-driven morphological change with neutral or negative fitness consequences cannot be resolved from the present data, as root ion content and spatial Na⁺ distribution were not assessed, and the pot conditions constrained soil volume and ion gradients.

The strong induction of *SOS1* (approximately 7-fold at 200 mM NaCl) in Sakha 5 is consistent with an increased reliance on the SOS pathway for Na⁺ extrusion, an energy-demanding process that requires sustained H⁺-ATPase activity^34,35^. Despite this induction, leaf K⁺ declined more in Sakha 5 than in Nubaria 1, suggesting that transcriptional activation of *SOS1* was not sufficient to maintain ion homeostasis at the leaf level under severe stress. Whether this reflects limitations in SOS pathway efficiency, continued Na⁺ uptake via other routes, or competitive displacement of K⁺ at the uptake stage cannot be distinguished without simultaneous measurements of Na⁺ flux and compartmentation.

The decline in net photosynthesis (Pₙ) across all cultivars was accompanied by a rise in intercellular CO₂ concentration (C_i_), from 327 to 364 µmol mol⁻¹ under control to 378–413 µmol mol⁻¹ at 200 mM NaCl, while stomatal conductance also declined. The combination of falling Pₙ and rising C_i_ indicates that the reduction in carbon fixation was not caused by CO₂ substrate limitation, pointing instead to non-stomatal constraints on photosynthetic capacity consistent with impaired Rubisco activity, reduced RuBP regeneration, or photosystem disruption under ionic stress^20^. Nubaria 1 showed a somewhat smaller proportional decline in Pₙ (55%) relative to its stomatal conductance reduction (67%), while Sakha 5 and Giza 716 showed proportionally larger photosynthetic losses. The degree to which these differences reflect differences in chloroplast ion balance, Rubisco stability, or other mesophyll-level factors remains to be determined^36,37^.

All three cultivars reached maximum epicuticular wax accumulation at 150 mM NaCl, followed by a decline at 200 mM NaCl in Sakha 5 and Giza 716; Nubaria 1 showed a smaller decline and maintained a relatively higher wax load under severe stress. In Sakha 5, *CER1* transcript levels continued to increase at 200 mM NaCl despite a decline in total wax, indicating that post-transcriptional or metabolic constraints limited wax biosynthetic output at the highest ionic load. One plausible constraint is competition for malonyl-CoA from the plastidial acetyl-CoA pool, which is shared between wax elongation and central carbon metabolism and may become limiting when photosynthesis is severely reduced^38,39^. The wax alcohol fraction showed the largest salt-induced increases in Sakha 5, consistent with induction of fatty acyl reductase activity, and changes in the alcohol-to-alkane ratio could affect cuticular water permeability independently of total wax load^40^.

The complete absence of pod set in Sakha 5 at 200 mM NaCl, compared with 1.78 pods per plant in Nubaria 1 under the same treatment, is among the most agronomically significant findings. Several non-exclusive factors may contribute: the 60% reduction in Pₙ at 200 mM NaCl would substantially reduce carbon availability to reproductive sinks; accumulation of Na⁺ and Cl⁻ in aerial tissues, including floral and pod tissues, could impair fertilization or early embryo development; and ionic stress is known to disrupt ABA and auxin signaling pathways that regulate reproductive development. The present data do not permit these possibilities to be distinguished, and targeted investigation of reproductive tissue ion content, carbon partitioning, and hormonal status under ionic stress would be needed to identify the primary cause.

The PCA showed that cultivar ellipses remained separated along PC1 across all salinity treatments, indicating that the traits driving cultivar identity, primarily root architecture in Sakha 5 and reproductive output in Nubaria 1, were not collapsed by stress into a common phenotype. The elongation of ellipses along PC2 with increasing NaCl reflects the within-cultivar variance attributable to stress severity. The stability of cultivar separation across treatments supports the use of root architecture and leaf K⁺ as phenotyping traits under saline conditions, as they reflect genotypic differences rather than stress-induced plasticity alone.

A key limitation of this study is the absence of leaf and root Na⁺ and Cl⁻ concentration data. The reviewer and editor have correctly identified this as a major gap. Direct quantification of Na⁺ and Cl⁻ accumulation in leaf and root tissues across all cultivar–treatment combinations is essential for evaluating whether the cultivar differences reported here reflect differential Na⁺ exclusion at the root level, differential compartmentation in leaf vacuoles, or a combination of both. These measurements would also allow calculation of K⁺/Na⁺ ratios and would permit mechanistic interpretation of the differential induction of *SOS1*, *HKT1*, and *NHX* class transporters observed across cultivars. We acknowledge that the interpretations offered in this manuscript regarding ion homeostasis are necessarily inferential in the absence of ionic content data and should be treated as working hypotheses to be tested in follow-up experiments incorporating ICP-OES analysis of Na⁺, K⁺, and Cl⁻ in both root and leaf fractions. Without these measurements, interpretations relating to ion exclusion, K⁺/Na⁺ discrimination, and the ionic phase of stress remain indirect and should be treated as hypotheses for future investigation. Additionally, the fold-change expression data reported here reflect induction relative to within-cultivar controls and do not capture differences in basal transcript abundance among cultivars, which may be substantial. Functional validation of the candidate regulatory associations suggested by co-expression patterns, particularly the co-induction of DREB2 with osmotic adjustment and antioxidant genes in Sakha 5, would require promoter-binding assays, time-course experiments, or loss-of-function studies.

## Conclusions

This study compared three Egyptian faba bean cultivars across three NaCl concentrations using an integrated set of morphological, physiological, biochemical, and gene expression measurements. The results showed that the cultivars differed in the pattern of their responses to ionic stress, not only in the overall degree of growth suppression. Nubaria 1 showed the best maintenance of leaf K⁺, net photosynthesis, and pod set under severe salinity (200 mM NaCl), combined with the lowest transcriptional induction across all gene categories. Sakha 5 showed the greatest transcriptional response, the largest stress-induced root elongation, and the strongest induction of proline biosynthesis, antioxidant, and ion-transport genes, but also the greatest reduction in reproductive output, including complete pod failure at 200 mM NaCl. Giza 716 was intermediate across most measures and had the highest net photosynthetic rate under control conditions. Across all cultivars, the simultaneous decline in net photosynthesis and rise in intercellular CO₂ under salinity indicated that non-stomatal limitation was the primary constraint on carbon fixation, rather than stomatal closure alone. Epicuticular wax accumulation peaked at moderate salinity in all cultivars and declined under severe stress despite continued *CER1* induction, suggesting that metabolic or post-transcriptional constraints limited wax biosynthesis at the highest NaCl level. Principal component analysis preserved cultivar separation across all salinity treatments, indicating that root architecture and reproductive investment are stable genotypic traits under ionic stress and are therefore tractable phenotyping targets.

## Supplementary Information

Below is the link to the electronic supplementary material.

Supplementary Material 1

## Data Availability

All data generated or analysed during this study are included in this published article. The gene sequences used for primer design are deposited in the National Center for Biotechnology Information (NCBI) GenBank database (https://www.ncbi.nlm.nih.gov/genbank/) under the following accession numbers: SOS1 (KF411441.1), HKT1 (HM584921.1), PIP2 (AJ748748.1), P5CS (EU267181.1), P5CR (XM_001506219.1), CAT (AJ250074.1), SOD (X54528.1), APX (AJ249351.1), DREB2 (KM114667.1), LEA (AJ557572.1), CER1 (XM_031428417.1), ACT (AJ507513.1), and UBQ (AB126078.1). Full primer sequences, amplicon sizes, and melting temperatures are provided in Supplementary Table S1. Additional datasets are available from the corresponding author (S.F.L.) upon reasonable request.

## References

[1] Basak, N. et al. *in Soil health and environmental sustainability: Application of geospatial technology* 107–129 (Springer, 2022).

[2] Harper, R., Dell, B., Ruprecht, J., Sochacki, S. & Smettem, K. *in Soils and landscape restoration* 193–208 (Elsevier, 2021).

[3] Hossain, A. et al. *in Environment, climate, plant and vegetation growth* 17–61 (Springer, 2020).

[4] Reed, M. S. & Stringer, L. C. *Land degradation, desertification and climate change: Anticipating, assessing and adapting to future change* (Routledge, 2016).

[5] Crépon, K. et al. Nutritional value of faba bean (Vicia faba L.) seeds for feed and food. *Field crops Res.* **115**, 329–339 (2010).

[6] Duc, G. Faba bean (Vicia faba L). *Field crops Res.* **53**, 99–109 (1997).

[7] Kislev, M. E. & Bar-Yosef, O. The legumes: the earliest domesticated plants in the Near East? *Curr. Anthropol.* **29**, 175–179 (1988).

[8] Zhang, H., Mascher, M., Abbo, S. & Jayakodi, M. Advancing grain legumes domestication and evolution studies with genomics. *Plant Cell Physiol.* **63**, 1540–1553 (2022). 35534441 10.1093/pcp/pcac062 PMC9680859

[9] Arnáez, J., Nadal-Romero, E., Lana-Renault, N. & García-Ruiz, J. M. Hydrological Challenges and Competing Demands in the Mediterranean Region. *Water Resour. Manage*. **40**, 96 (2026).

[10] Tosoroni, A. Pre-breeding tools: bridging conservation, utilization and valorization of food legumes. (2025).

[11] Ma, L., Liu, X., Lv, W. & Yang, Y. Molecular mechanisms of plant responses to salt stress. *Front. Plant Sci.* **13**, 934877 (2022). 35832230 10.3389/fpls.2022.934877 PMC9271918

[12] Balasubramaniam, T., Shen, G., Esmaeili, N. & Zhang, H. Plants’ response mechanisms to salinity stress. *Plants* **12**, 2253 (2023). 37375879 10.3390/plants12122253 PMC10300796

[13] Verma, O. et al. Salinity stress effect on staple food crops and novel mitigation strategies. *Biologia* **79**, 2359–2374 (2024).

[14] Fauzia, A. N. & Anisa, N. F. Molecular Physiological Characterization of Tissue Tolerance in Rice under Salt Stress.

[15] Tavakkoli, E., Watts-Williams, S. J., Rengasamy, P. & McDonald, G. K. Eliciting the aboveground physiological regulation that underlies salinity tolerance in faba bean (Vicia faba L). *Environ. Exp. Bot.* **226**, 105849 (2024).

[16] Parisa, D., Sagervanshi, A., Abdalla, M. A. & Mühling, K. H. Na⁺/Mg²⁺ ratio: a new physiological trait for salt resistance in faba bean (Vicia faba L). *BMC Plant Biol.* **25**, 1568 (2025). 41239236 10.1186/s12870-025-07698-x PMC12616959

[17] Liu, H. et al. Signaling transduction of ABA, ROS, and Ca2 + in plant stomatal closure in response to drought. *Int. J. Mol. Sci.* **23**, 14824 (2022). 36499153 10.3390/ijms232314824 PMC9736234

[18] Huang, S. et al. Under salt stress, quinoa stomatal guard cells control transpiration in an ABA-primed manner. *New Phytol.* **249**, 2372–2385 (2026). 41413957 10.1111/nph.70853

[19] Bosque, H. & Rodríguez, J. P. *in Biology and Biotechnology of Quinoa: Super Grain for Food Security* 195–220 (Springer, 2022).

[20] Pan, T. et al. Non-stomatal limitation of photosynthesis by soil salinity. *Crit. Rev. Environ. Sci. Technol.* **51**, 791–825 (2021).

[21] Dehghanian, Z. et al. Quinoa: a promising crop for resolving the bottleneck of cultivation in soils affected by multiple environmental abiotic stresses. *Plants* **13**, 2117 (2024). 39124236 10.3390/plants13152117 PMC11313704

[22] Rasouli, F., Kiani-Pouya, A., Zhang, H. & Shabala, S. *in Biology and Biotechnology of Quinoa: Super Grain for Food Security* 221–242 (Springer, 2022).

[23] Ma, Y., Wang, Z., Zhou, B., Yang, W. & Wang, Y. Salicylic acid improving salinity tolerance by enhancing photosynthetic capacity, osmotic adjustment and maintenance of Na+/K+ homeostasis in faba bean seedlings. *Chem. Biol. Technol. Agric.* **12**, 89 (2025).

[24] El-Sayed, A. F., Abou-Zeid, A., Kandeel, D. M. & Sallam, A. Evaluation of Some Faba Bean Genotypes Under Soil Salinity in Nubaria Region, Egypt. *J. King Abdulaziz University: Meteorol. Environ. Arid Land. Agric.* **34**, 161–172 (2025).

[25] Kumar, A. & Lorand, D. Robust ∆∆ct estimate. *Genomics* **113**, 420–427 (2021). 33309766 10.1016/j.ygeno.2020.12.009

[26] Munns, R. & Tester, M. Mechanisms of salinity tolerance. *Annu. Rev. Plant. Biol.* **59**, 651–681 (2008). 18444910 10.1146/annurev.arplant.59.032607.092911

[27] Mulet, J. M., Porcel, R. & Yenush, L. Modulation of potassium transport to increase abiotic stress tolerance in plants. *J. Exp. Bot.* **74**, 5989–6005 (2023). 37611215 10.1093/jxb/erad333

[28] Mostofa, M. G. et al. Potassium in plant physiological adaptation to abiotic stresses. *Plant Physiol. Biochem.* **186**, 279–289 (2022). 35932652 10.1016/j.plaphy.2022.07.011

[29] Shabala, S. & Pottosin, I. Regulation of potassium transport in plants under hostile conditions: implications for abiotic and biotic stress tolerance. *Physiol. Plant.* **151**, 257–279 (2014). 24506225 10.1111/ppl.12165

[30] Han, H., Adamowski, M., Qi, L., Alotaibi, S. S. & Friml, J. PIN-mediated polar auxin transport regulations in plant tropic responses. *New Phytol.* **232**, 510–522 (2021). 34254313 10.1111/nph.17617

[31] Lee, H. J., Kim, H. S., Park, J. M., Cho, H. S. & Jeon, J. H. PIN-mediated polar auxin transport facilitates root– obstacle avoidance. *New Phytol.* **225**, 1285–1296 (2020). 31336402 10.1111/nph.16076

[32] Aufar, M. & Azwar, K. Morphological and Physiological Responses of Crop Plants to Salinity Stress: A Systematic Review. *Contrib. Cent. Res. Inst. Agric.* **20**, 44–53 (2026).

[33] Srivastava, D. et al. *in Decoding Plant–Environment–Microbiome Interactions in Stress-Resilient Agriculture* 65–79 (Elsevier, 2026).

[34] Silva, P. & Gerós, H. Regulation by salt of vacuolar H+-ATPase and H+-pyrophosphatase activities and Na+/H+ exchange. *Plant Signal. Behav.* **4**, 718–726 (2009). 19820346 10.4161/psb.4.8.9236 PMC2801382

[35] Batelli, G. et al. SOS2 promotes salt tolerance in part by interacting with the vacuolar H+-ATPase and upregulating its transport activity. *Mol. Cell. Biol.* **27**, 7781–7790 (2007). 17875927 10.1128/MCB.00430-07 PMC2169139

[36] Gan, P., Liu, F., Li, R., Wang, S. & Luo, J. Chloroplasts—beyond energy capture and carbon fixation: tuning of photosynthesis in response to chilling stress. *Int. J. Mol. Sci.* **20**, 5046 (2019). 31614592 10.3390/ijms20205046 PMC6834309

[37] Singh, S. K. & Reddy, V. R. Co-regulation of photosynthetic processes under potassium deficiency across CO2 levels in soybean: mechanisms of limitations and adaptations. *Photosynth. Res.* **137**, 183–200 (2018). 29478203 10.1007/s11120-018-0490-3

[38] Yang, X. et al. Acetyl-CoA Carboxylase1 influences ECERIFERUM2 activity to mediate the synthesis of very-long-chain fatty acid past C28. *Plant Physiol.* **197**, kiae253 (2025). 38709681 10.1093/plphys/kiae253

[39] Zheng, H., Rowland, O. & Kunst, L. Disruptions of the Arabidopsis enoyl-CoA reductase gene reveal an essential role for very-long-chain fatty acid synthesis in cell expansion during plant morphogenesis. *Plant. Cell.* **17**, 1467–1481 (2005). 15829606 10.1105/tpc.104.030155 PMC1091768

[40] Li, S. et al. Deciphering the core shunt mechanism in Arabidopsis cuticular wax biosynthesis and its role in plant environmental adaptation. *Nat. Plants*. **11**, 165–175 (2025). 39753959 10.1038/s41477-024-01892-9

