# Supplementary material for: Integrating morphophysiological traits with salt-responsive gene expression uncovers cultivar-specific tolerance mechanisms in faba beans facing NaCl stress
Source: Sci Rep. 2026 May 10;16:14702. doi: 10.1038/s41598-026-51413-1 (PMC13158290; doi:10.1038/s41598-026-51413-1)
Supplement: Supplementary file 1 — Supplementary Material 1 [file 41598_2026_51413_MOESM1_ESM.docx]

**Table S1.** Gene-specific primers used for quantitative RT-PCR analysis of salt-responsive and reference genes in *Vicia faba* L.

| **Gene** | **Full name / Function** | **Dir.** | **Primer sequence (5′→3′)** | **Tm (°C)** | **Amp. (bp)** | **Accession** |
| --- | --- | --- | --- | --- | --- | --- |
| ***SOS1*** | Salt Overly Sensitive 1 \| Ion transport | **F** | GCTGCTGAAGATGGCGTTCTTG | 60.8 | 147 bp | KF411441.1 |
|  |  | **R** | ACACCAGCTTTGGTCATCAGCA | 60.4 |  |  |
| ***HKT1*** | High-Affinity K⁺ Transporter 1 \| Ion transport | **F** | TGGTGGCAATCTCATCGTCTTC | 59.6 | 118 bp | HM584921.1 |
|  |  | **R** | CGAAGAACAGCATCAGCACCAG | 60.2 |  |  |
| ***PIP2*** | Plasma Membrane Intrinsic Protein 2 \| Aquaporin | **F** | ATGGCTGCAGAGAACAACGAGG | 61.0 | 162 bp | AJ748748.1 |
|  |  | **R** | TCAGCACCAGTAGAAGCCACCA | 60.6 |  |  |
| ***P5CS*** | Δ¹-Pyrroline-5-Carboxylate Synthetase \| Proline biosynthesis | **F** | GCACAAGATGCTGCCAGAGATG | 60.4 | 134 bp | EU267181.1 |
|  |  | **R** | TCCATCACCTGCTTCTTGACCG | 60.8 |  |  |
| ***P5CR*** | Pyrroline-5-Carboxylate Reductase \| Proline biosynthesis | **F** | GGAGGAGATGGCAGAGAAGACC | 60.2 | 158 bp | XM_001506219.1 |
|  |  | **R** | ACCTTGAGCTTGGTCTTGGCAG | 60.6 |  |  |
| ***CAT*** | Catalase \| ROS scavenging | **F** | TGGACAAGGCTCAGCAGAAGTC | 60.0 | 121 bp | AJ250074.1 |
|  |  | **R** | CCAGCATCTCCACCATCACCTT | 60.4 |  |  |
| ***SOD*** | Cu/Zn-Superoxide Dismutase \| ROS scavenging | **F** | AAGCCTGACGGAGACAACAAGG | 60.6 | 145 bp | X54528.1 |
|  |  | **R** | TGCCACCAATGAGACCAGCAAT | 59.8 |  |  |
| ***APX*** | Ascorbate Peroxidase \| ROS scavenging | **F** | TCGTCAACACCAAGCCTCTCAC | 60.4 | 107 bp | AF221antler → AJ249351.1 |
|  |  | **R** | AGCAACACCGTCAGCATCAAAG | 59.6 |  |  |
| ***DREB2*** | DREB2 Transcription Factor \| Stress regulon activator | **F** | CAGCAGCAGGAGAAGGAGAAGG | 60.8 | 176 bp | KM114667.1 |
|  |  | **R** | TGCACCTTGTTCTCCTCGTCCT | 61.0 |  |  |
| ***LEA*** | Late Embryogenesis Abundant Protein \| Stress protection | **F** | GCCGACAAGGAGGAGAAGAAGG | 61.0 | 138 bp | AJ557572.1 |
|  |  | **R** | AGCACCTTCTTGCCCTCAGACT | 60.4 |  |  |
| ***CER1*** | ECERIFERUM 1 \| Very-long-chain alkane biosynthesis | **F** | TGTGGTGGCAATCTTCGTCTTG | 59.8 | 193 bp | XM_031428417.1 |
|  |  | **R** | AGCCACAGCAACCAAGACCTTC | 60.6 |  |  |
| **Reference genes** | | | | | | |
| ***ACT*** | Actin \| Reference gene | **F** | TGAGCACAATGTTACCGTCAGG | 59.4 | 112 bp | AJ507513.1 |
|  |  | **R** | GGATAGCATGGAGGAAGACAGC | 59.8 |  |  |
| ***UBQ*** | Ubiquitin \| Reference gene | **F** | GCAAGACCAAGAGCAAGAGACC | 59.6 |  | AB126078.1 |
|  |  | **R** | TGACCAGCAAGGATGAGAACCC | 60.0 | 124 bp |  |
